# Epithelioid Type Tongue Rhabdomyosarcoma in a Pediatric Patient: A Case Report With Literature Review

**DOI:** 10.7759/cureus.30820

**Published:** 2022-10-29

**Authors:** Baraa I Awad, Dania A Kouther, Mohammed Al-Garni

**Affiliations:** 1 Department of Otolaryngology-Head & Neck Surgery, King Saud Bin Abdulaziz University for Health Sciences College of Medicine, Jeddah, SAU; 2 Department of Otolaryngology-Head & Neck Surgery, King Abdullah International Medical Research Center, Jeddah, SAU; 3 Department of Otolaryngology-Head & Neck Surgery, Ministry of National Guard Health Affairs, Jeddah, SAU

**Keywords:** epithelioid, immunohistochemistry, embryonal, oral cavity, tongue, rhabdomyosarcoma

## Abstract

Tongue rhabdomyosarcomas (RMSs) are extremely rare soft tissue tumors in the pediatric age group. The most common reported histopathology type is embryonal. To our knowledge, epithelioid type has not yet been reported in tongue RMSs. We report a case of an eight-year-old boy who presented with a painless tongue mass, and the biopsy demonstrated RMS epithelioid type. Head magnetic resonance imaging (MRI) was performed and showed peripherally enhancing mass with central cystic/necrotic component. Computed tomography (CT) scan of the neck showed involvement of the cervical lymph nodes, while metastatic workup was negative for malignancy. As the biopsy showed a positive margin, the patient underwent secondary resection. Moreover, he received adjuvant chemotherapy and radiotherapy. There was no evidence of the disease, and no metastasis was detected in a follow-up of three years. We also performed a literature review of pediatric tongue RMSs to assess the clinical presentation, histopathology, diagnosis, and management.

## Introduction

Rhabdomyosarcomas (RMSs) are malignant tumors that originate from primitive mesenchymal tissue. They are the most common soft tissue tumor in children [1]. Soft tissue tumors are responsible for 3 to 4% of all tumors in the pediatric age group. Approximately, half of all soft tissue tumors are RMSs [2,3]. Two-thirds of the cases reported to have RMSs are younger than six years of age, with a male-to-female ratio between 1.3 to 1.5 [2].

The etiology and specific risk factors of RMSs are unknown; however, higher incidence was observed in children from families with low socioeconomic status, children exposed to radiation in utero, and with maternal use of recreational drugs [4-6]. RMSs can arise from different sites of the body. In children, 35% of RMSs are located in the head and neck. Among head and neck RMSs, 30% originated in oral and pharyngeal structures [1]. The tongue, however, is an uncommon site for this tumor. The clinical features of RMSs vary according to the site and stage. Generally, a painless mass without associated symptoms is the main presentation of tongue RMSs [7].

The differential diagnosis that should be considered includes other soft tissue tumors such as hemangioma, fibroma, rhabdomyoma, and lymphangioma [7]. To evaluate the primary site of the tumor with its surrounding structures, computed tomography (CT) and magnetic resonance imaging (MRI) with contrast are done. These aid in generating a differential diagnosis of the mass. To initially diagnose RMSs, total removal of the tumor with negative margins should be performed. Incisional biopsy, on the other hand, should be done if resection will affect the function. The definitive diagnosis is based on histopathological evidence of myogenesis, strap or tadpole cells, and individual tumor cells with or without cross-striation [1]. As RMSs are usually poorly differentiated, the diagnosis is often difficult. RMSs can be classified based on histopathology and molecular pathology into: (a) embryonal RMS; (b) botryoid and spindle cell RMS; (c) alveolar RMS, and (d) undifferentiated sarcoma. The majority of oral RMSs are embryonal [1].

For staging, chest CT scan, bone marrow aspiration and bone scan can also be performed. However, the metastatic workup should be tailored to the clinical and histologic features. Clinical group and the tumor, node, metastasis (TNM) system are the most widely used classifications for newly diagnosed RMSs. The clinical group (CG) takes into account the tumor size, nodal involvement, and patient age. It has four categories, which take into account the extent of the disease [8].

The treatment of oral RMSs consists of surgery followed by chemotherapy with or without radiotherapy. Chemoradiation following complete surgical resection is considered ideal, however, it is difficult to achieve complete excision. Oral RMSs are associated with a higher recurrence rate and metastasis compared to other types. With the multimodal treatment approach, the five-year survival rate reaches to 85% [1].

We report a case of an eight-year-old male who presented with a small mass at the tip of the tongue that was excised with positive margins. He underwent a second resection of the mass with neck dissection and received adjuvant chemotherapy and radiation therapy. We also reviewed reported cases of tongue RMSs in the literature on the clinical presentation, treatment, and outcomes.

## Case presentation

An eight-year-old child presented to the referring hospital with a small swelling at the tip of his tongue noticed by his parents. According to medical records, there were no associated symptoms and signs such as pain, difficulty feeding, fever, or lymph node enlargement at the time of presentation. The patient had a family history of cancer in parents, aunts, and grandparents (acute lymphocytic leukemia, breast, brain, and prostate cancer).

**Figure 1:**
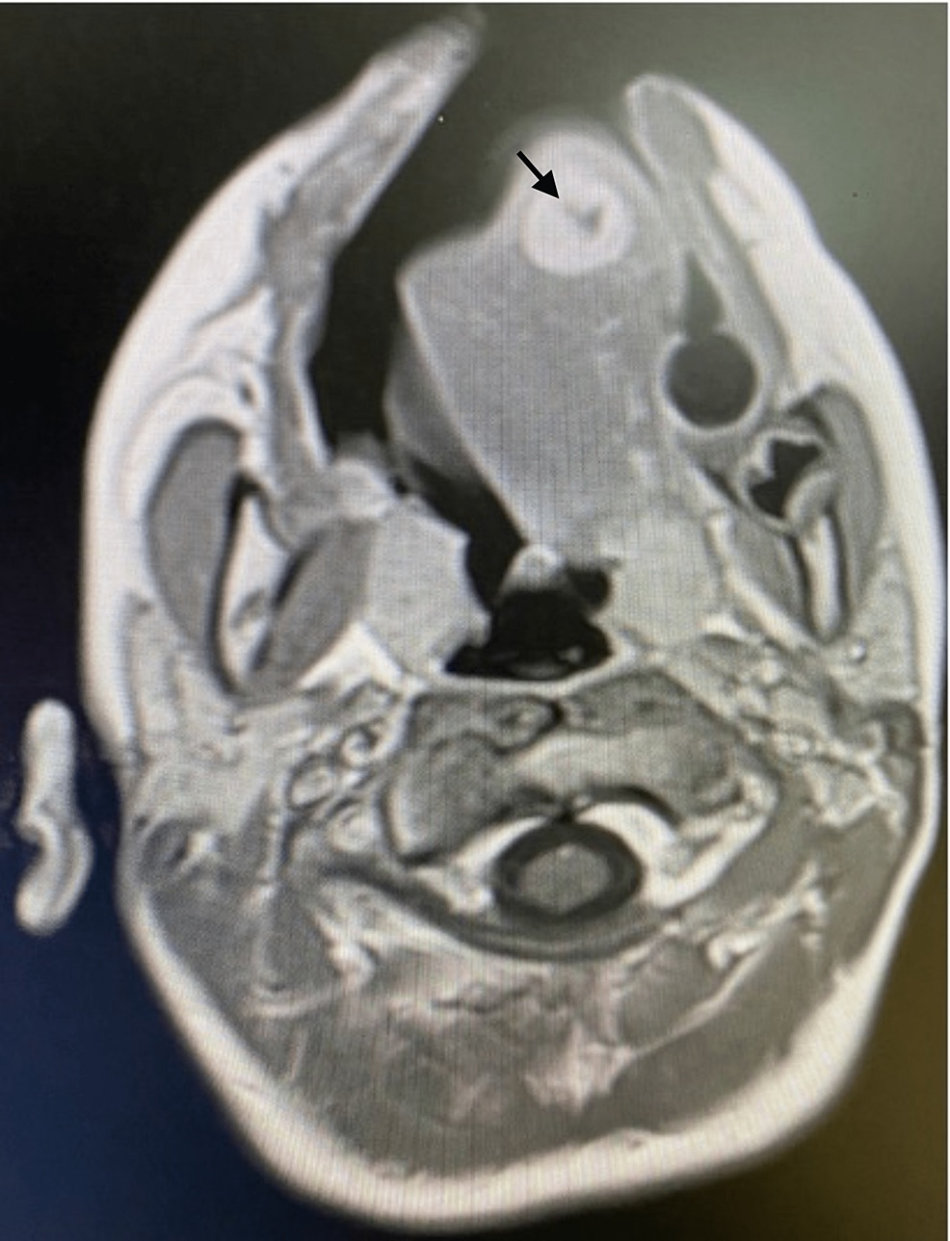
Axial T1 post-contrast magnetic resonance imaging (MRI) of the head demonstrates a peripherally enhancing mass measuring 1.3 x 1.4 cm (black arrow) with central cystic/necrotic component.

**Figure 2:**
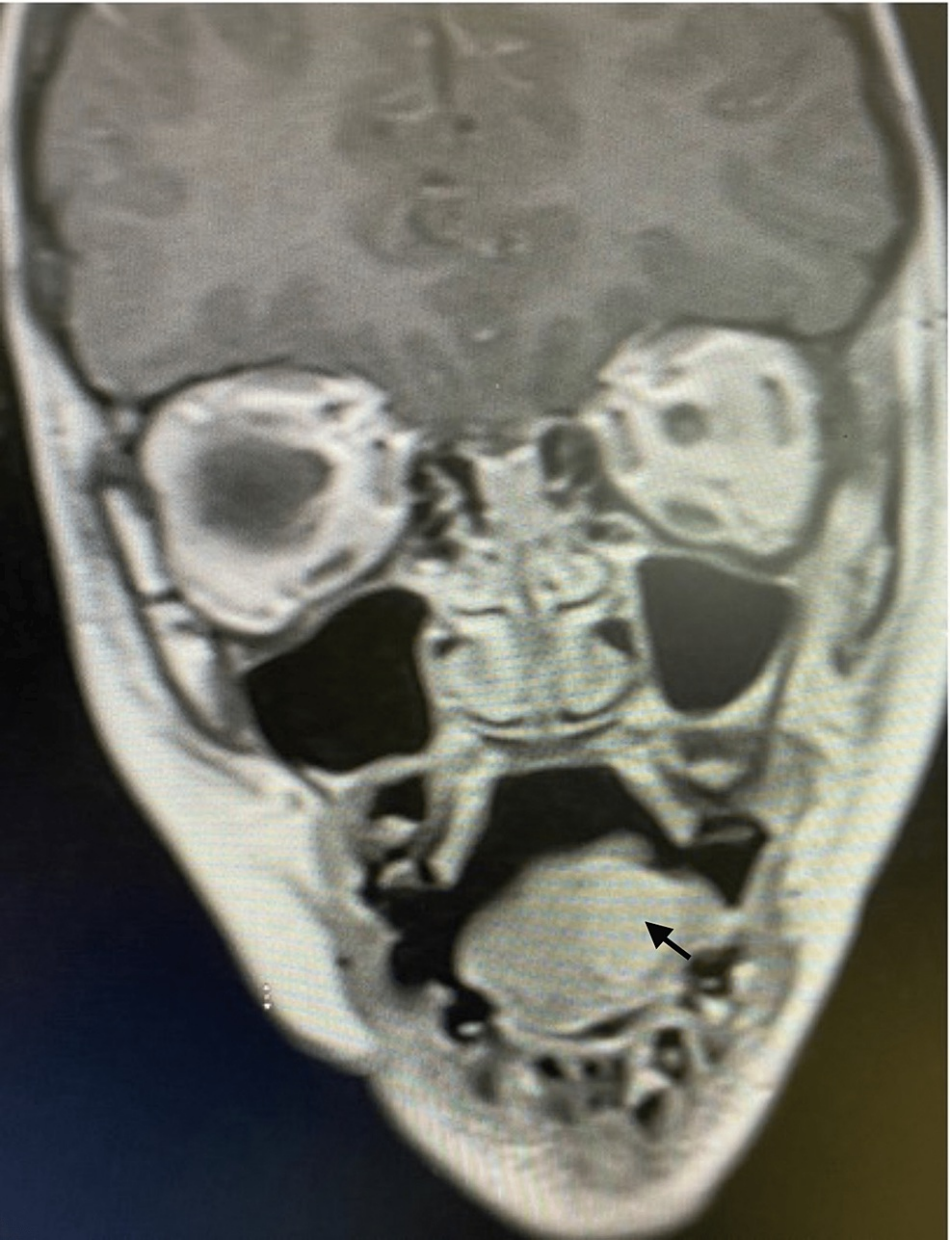
Coronal T1 post-contrast magnetic resonance imaging (MRI) of the head demonstrates the cranial caudal dimension of the enhancing lesion (black arrow) measuring 1.7 cm with no surrounding edema or regional lymphadenopathy.

Head magnetic resonance imaging (MRI) was performed before the patient was referred to our hospital. The medical records reported a 1.3 x 1.4 x 1.7 cm peripherally enhancing mass with central cystic/necrotic component (Figures 1, 2). The patient subsequently underwent an excision biopsy of the mass. The excised mass was reported as 4 x 1 cm. According to the previous reports, histopathology consisted of epithelioid rhabdomyosarcoma with the tumor present at the excision margins.

As a result, the patient was referred to our hospital for further management. Oral cavity examination revealed no swelling of the tongue, and only a healed operational scar was noted. All head and neck lymph nodes were non-palpable on clinical exam. Other organ systems examination was unremarkable. Head and neck computed tomography (CT) scan demonstrated an oral cavity filled with heterogeneous tongue without bone destruction at the level of the neck. Also, bilateral enlarged cervical lymph nodes at the left level I were noted and the largest measured 0.8 x 1.1 cm in the left cervical chain. Oropharynx, hypopharynx, and major cervical vessels appeared unremarkable. The larynx and trachea were patent. CT scan (chest, abdomen, and pelvis), bone marrow aspiration, and bone scan were all negative for metastasis. As the patient had a strong family history of cancer, whole exome sequencing (WES) was performed, and it was negative. A tumor board meeting consisting of an otolaryngology and head and neck surgeon, pediatric surgeon, radiotherapist, pediatric otolaryngology surgeon, histopathologist, radiation oncologist and pediatric medical oncologist was conducted. The members of the tumor board agreed on doing secondary resection. If the margins were negative, the patient will be treated as intermediate risk.

**Figure 3:**
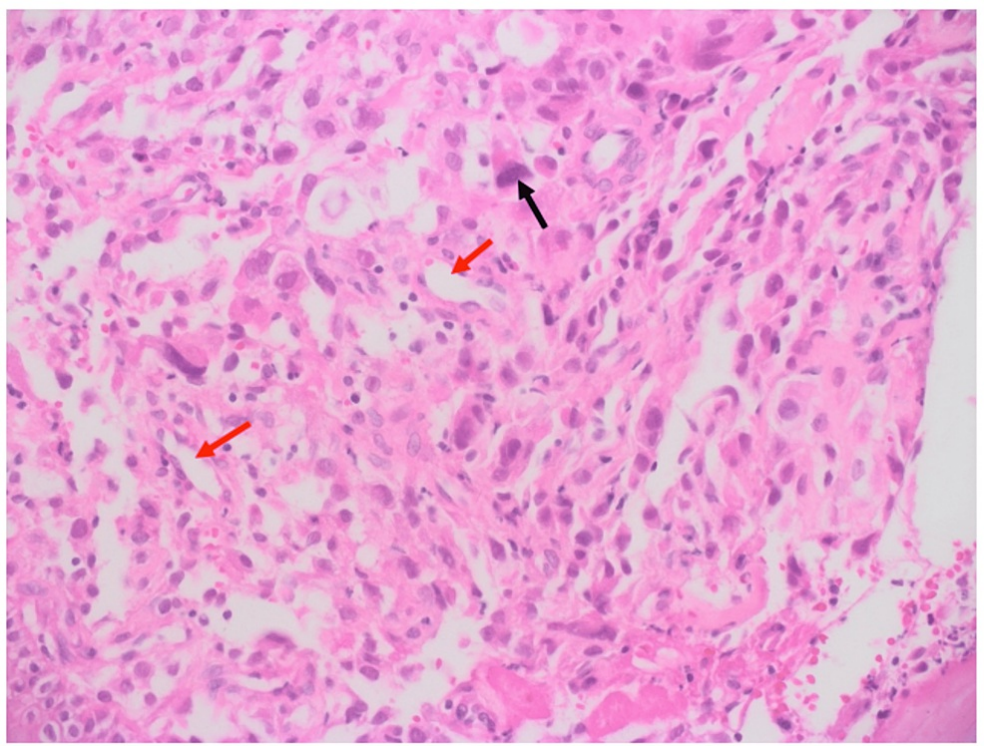
Histopathology examination with hematoxylin and eosin (H&E) stain demonstrated diffuse growth of epithelioid neoplastic cell (black arrow) with small vessels (red arrow) (magnification, x20).

**Figure 4:**
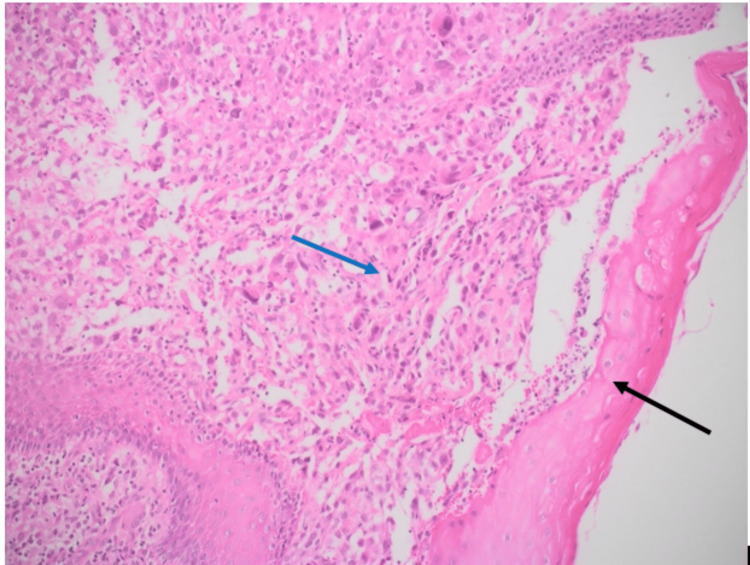
Histopathology examination with hematoxylin and eosin (H&E) stain showed a neoplasm with diffuse solid growth consisting of typical epithelioid cell (blue arrow) with hyperchromatic nuclei and high nuclear/cytoplasmic ratio overlying normal tongue mucosa (black arrow) (magnification, x10).

**Figure 5:**
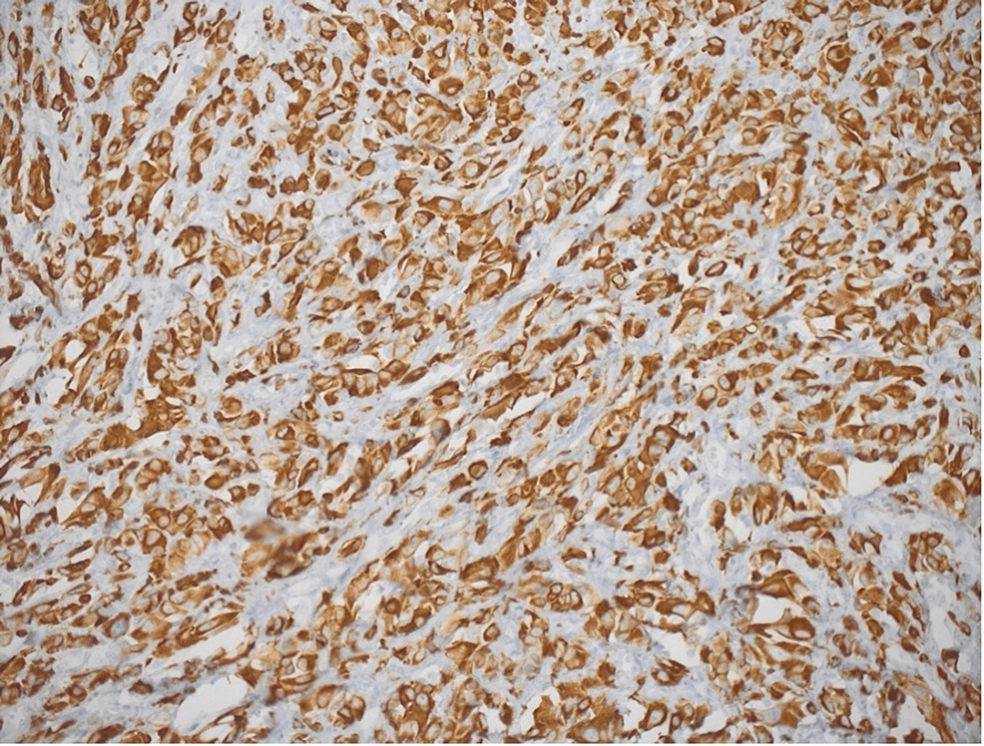
Immunohistochemistry study showed diffuse positivity of desmin confirming the muscular origin of the neoplasm (magnification, x10).

The patient underwent wedge resection of the tongue with left neck dissection from level I to level III lymph nodes. Wider resection of the old lesion with resection of the new lesion was performed with a 0.5 cm margin from the mass. Negative margins were reported by a frozen section procedure intraoperatively. Primary closure of the defect was then done. Histopathology report showed epithelioid RMS, Grade 2, with a tumor size of 0.8 x 0.5 x 0.5 cm that extended deeply in the tongue tissue. The tumor pathological stage (pTNM) was pT1b, pN1. Histopathological analysis of the hematoxylin and eosin-stained material demonstrated diffuse growth of epithelioid neoplastic cells with hyperchromatic nuclei and high nucleus-cytoplasm ratio (Figures 3, 4). Resection margins were all negative. In addition, necrosis, lymphovascular invasion, and perineural invasion were not identified. The neoplastic cells showed strong and diffuse positivity for desmin on immunohistochemistry (Figure 5), confirming muscular histotype origin of the neoplasm. Lymph node biopsy showed two out of 33 were positive for malignancy. For staging, we used clinical grouping which takes into account the tumor size, nodal involvement, and patient age. It has four categories (CG I to IV) as demonstrated in Table 1 [8]. Accordingly, the tumor staged T1, N1, M0, and clinical group 1 stage II with intermediate risk.

**Table 1:** Clinical grouping of RMS by the intergroup rhabdomyosarcoma study group (IRSG) RMS: Rhabdomyosarcoma

Clinical Group (CG)	Extent of the disease
I	Localized tumor, complete resection
II	Microscopic residual disease
III	Gross residual disease
IV	Distant metastasis

Post-operative MRI revealed a residual area of abnormal signal with enhancement at the region of the surgical resection of the tip of the tongue on the left side, which was highly suggestive of post-surgical changes. Residual small lymph nodes at level II of the cervical area and left prevertebral region were noted as well.

Postoperatively, the patient received the Children’s Oncology Group (COG) ARST 0531 chemotherapy protocol which consists of vincristine, actinomycin d and cyclophosphamide (VAC) alternating with vincristine and irinotecan (VI) for 40 weeks. He also completed radiotherapy to oral anterior tongue, bilateral cervical and retropharyngeal lymph nodes with a dose of 60 Gy in 40 fractions. During his follow-up for the last three years in our hospital, no evidence of the disease was noted in the clinical and radiological assessment done by serial MRIs.

## Discussion

We reviewed all the cases reported in the English language on tongue RMSs in pediatrics using the PubMed engine. Twenty cases were reported, including our case, summarized in Table 2 [7, 9-26]. Ten (50%) of the cases were female. The median age at diagnosis was three months ranging from prenatally diagnosed to eight years, with an average of 21 months. The clinical presentation varied among the reported cases. Eleven patients presented initially with painless tongue mass, and only one patient presented with enlarged lymph nodes. Others presented with either tongue bleeding or difficulty in sucking. Among RMS types, embryonal was the most common as it has been reported in 14 cases. However, epithelioid type has never been reported in tongue RMS. Surgical excision alone was performed in five cases, and surgery plus chemotherapy and radiation were performed in five cases as well. In eight cases, the management was surgery combined with chemotherapy. Most of the cases reported no evidence of disease or metastasis during long-term follow-up. Only one patient who underwent surgical excision plus chemotherapy had lung metastasis.

**Table 2:** Cases of tongue RMSs in pediatrics RMS: Rhabdomyosarcoma

Case	Study (Year) [Reference]	Sex	Age at diagnosis (Months)	Presentation	Histology	Treatment	Prognosis
1	Liebert and Stool (1973) [9]	Male	3	Mass at the base of the tongue, cough difficulty sucking	Embryonal	Excision biopsy, Chemotherapy, Radiotherapy	5 years: no evidence of disease
2	Bras et al. (1987) [10]	Female	83.99	-	Embryonal	Excision biopsy, Chemotherapy, Radiotherapy	1 year: no evidence of disease
3	Gupta et al. (1990) [11]	Female	0.066	Mass of the tongue since birth	Congenital embryonal	Surgery "excision and repair"	1.5 months: no evidence of disease
4	Kodet et al. (1991) [12]	Female	40	Second recurrence after rhabdomyoma, tumor mass was found at the left base of the tongue with extension to the adjacent tonsil and its bed	Mixed embryonal/ alveolar	Surgery, radiotherapy, chemotherapy	47 months: no evidence of disease
5	Mohan and Lal (1992) [13]	Female	0.066	Mass arising from the anterior 2/3 of the tongue	Congenital embryonal	Surgery "excision and repair"	-
6	Nag et al. (1993) [14]	Female	3	Mass in the left posterior part of the tongue	Embryonal	Chemotherapy, Radiotherapy	38 months: no evidence of disease
7	Doval et al. (1994) [15]	Male	42	Swelling of the tongue	Alveolar	Chemotherapy, surgery partial glossectomy"	84 months: no evidence of disease
8	Skelton and Goodwin (1999) [16]	Male	0	Prenatal US: mass at the base of the tongue	Embryonal	Surgery, chemotherapy	8 months of chemotherapy: partial response
9	van Grotel et al. (2003) [17]	Male	4	Rapidly growing swelling of the tongue, sucking problems, mild airway obstruction	Embryonal	Chemotherapy, brachytherapy, surgery "partial glossectomy*	3 years: no evidence of disease
10	Gupta et al. (2004) [18]	Female	0.099	Congenital lesion involves right base of the tongue and mouth floor.	Spindle cell	Chemotherapy, surgery	3 months: residual tumor
11	Cirocco et al. (2005) [19]	Female	10	Erythematous nodule located on the distal portion of the tongue	Embryonal	Chemotherapy, surgery "partial glossectomy"	30 months: lung metastasis
12	Gupta et al. (2006) [20]	Male	24	Redish lesion on the right side of dorsum of the tongue with bleeding	Embryonal	Surgery "near total excision of the mass", Chemotherapy	-
13	Childs and Goudy (2010) [21]	-	0.066	Tongue mass	Embryonal	Chemotherapy, surgery "near total glossectomy + excision of submandibular and sublingual glands"	-
14	Kebudi and Ozdemir (2010) [7]	Male	24	Persistent mass after tongue biting	Embryonal	Surgery. chemotherapy	6 months: no evidence of disease
15	Gurung et al. (2012) [22]	-	0	Tongue mass arising from the dorsal surface of anterior 2/3 of tongue	Botryoid	Excision of the mass, chemotherapy, radiotherapy	-
16	Soomro and Mughal (2014) [23]	Female	0.033	Mass of the tongue extending from its middle 1/3 to posterior 1/3. The antenatal history was insignificant. The baby was unable to take feeds orally.	Embryonal	Excision, chemotherapy	-
17	Chatopadhayay et al. (2016) [24]	Male	0.822	Mass arising from anterior 2/3 of the tongue, respiratory distress, choanal atresia	Embryonal	Surgery mass excision"	3 years: no evidence of disease
18	Balasundaram et al. (2016) [25]	Female	0	Swelling with ulceration at the tip of the tongue, and bleeding	Spindle cell	Excision of the mass	-
19	Oudrhiri et al. (2019) [26]	Female	95.99	Mass of the oral cavity gradually increasing in volume causing discomfort to the mobilization of the tongue, snoring and sometimes deglutition disorders. mass extending on the left edge of the tongue with infra-centimeter and sub-mandibular cervical lymphadenopathy.	Embryonal	Chemotherapy, surgery	1 year: no evidence of disease
20	Current study	Male	95.99	Small swelling at the tip of the tongue	Epitheliod	Surgery, radiotherapy, chemotherapy	3 years: no evidence of disease

RMSs of the tongue are rare. Bras et al. reported 16 cases of oral RMSs between 1944 to 1984, only one case has tongue RMSs [10]. The most frequently reported histopathology type is embryonal. Our patient had epithelioid type of tongue RMSs, which to our knowledge, has not been reported before in literature. Tongue RMSs initially present as a painless tongue mass. In almost all cases, RMSs have been detected early in the disease course. To our knowledge, only one case was reported by Oudrhiri, in which the patient presented with submandibular and cervical lymphadenopathy in clinical examination [26]. In our case, lymph nodes clinically were not palpable, while CT scan showed cervical lymph nodes enlargement.

Treatment of tongue RMSs is multimodal including surgery plus chemotherapy, chemotherapy plus radiotherapy, and triple therapy (surgery, chemotherapy, and radiotherapy). Chemotherapy has been used in all the cases as RMSs are chemosensitive, while surgery is considered mainly in patients with respectable tumors. Surgical resection includes excisional biopsy, partial glossectomy, and near total glossectomy depending on tumor size. Radiotherapy is reserved for the control of the locally advanced disease. In the case reviewed by Gurung et al., the patient received radiotherapy along with surgery and chemotherapy as histopathology showed invasion to the tongue muscle [14]. Similarly to our patient, triple therapy was considered as the tumor was deeply extended in the tongue tissue along with positive lymph nodes. However, Oudrhiri’s case had lymph nodes involvement and the patient did not receive radiation and was treated with surgical resection and adjuvant chemotherapy [18]. Both patients showed an excellent response, and no evidence of the tumor was detected in one-year follow-up. Generally, the prognosis of RMSs has improved to 65% as compared to what it was in the 1960s (10%). A more favorable prognosis (80%) is found in stage I lesions [1]. Although metastasis was found in our patient, it is rarely reported in literature.

## Conclusions

RMSs of the tongue are a rare condition with very few cases reported in the literature. The most common presentation is a lingual mass, and due to its location, it is identified and diagnosed early in the course of the disease. The most common RMS is embryonal and to the best of our knowledge, to date there are no cases reported on epitheliod RMS. Treatment depends on the stage and involves surgical resection, chemotherapy, and radiation.
